# Peptide Nanoarray Scaffold Vaccine for SARS-COV-2 and Its Variants of Concerns

**DOI:** 10.21203/rs.3.rs-1206402/v1

**Published:** 2022-01-24

**Authors:** Karen Zagorski, Kabita Pandey, Rajesh Rajaiah, Omalla Olwenyi, Aditya Bade, Arpan Acharya, Morgan Johnston, Shaun Filliaux, Yuri Lyubchenko, Siddappa Byrareddy

**Affiliations:** University of Nebraska Medical Center

**Keywords:** variants of concerns (VOC), SARS-CoV-2, peptide, vaccine, PADRE, epitope, mutations, vaccines

## Abstract

The current vaccine development strategies for the COVID-19 pandemic utilize whole inactive or attenuated viruses, virus-like particles, recombinant proteins, and antigen-coding DNA and mRNA with various delivery strategies. While highly effective, these vaccine development strategies are time-consuming and often do not provide reliable protection for immunocompromised individuals, young children, and pregnant women. Here, we propose a novel modular vaccine platform to address these shortcomings using chemically synthesized peptides and identified based on the validated bioinformatic data about the target. The vaccine is based on the rational design of an immunogen containing two defined B-cell epitopes from the spike protein of SARS-Co-V2 and a universal T-helper epitope PADRE assembled on the DNA scaffold. The results demonstrate that this assembly is immunogenic and generates neutralizing antibodies against SARS-CoV-2 wild type and its variants of concerns (VOC). This newly designed peptide nanoarray scaffold vaccine is useful in controlling virus transmission in immunocompromised individuals, as well as individuals who are prone to vaccine-induced adverse reactions. Given that the immunogen is modular, epitopes or immunomodulatory ligands can be easily introduced in order to tailor the vaccine to the recipient. This also allows the already developed vaccine to be modified rapidly according to the identified mutations of the virus.

## Introduction

The ongoing SARS-CoV-2 and its variants of concerns (VOCs) pandemic has demonstrated the need for vaccine technologies capable of ensuring rapid and efficient response to novel pathogens. Current vaccines for SARS-CoV-2 are extremely effective^1–5^, but their inflammatory nature limits the use in individuals with immune disorders^6–12^. Moreover, herd immunity towards novel pathogens has proven unreliable and difficult to achieve, leaving immunocompromised individuals at risk^13,14^. Immunocompromised individuals are at greater risk of infection and may remain infectious for longer than the immunocompetent, further increasing the rate of community spread^15–17^. This highlights the need for vaccine platforms that can be individualized and adapted for immunocompromised individuals and individuals with autoimmune disorders^18^. Vaccine development, production, and distribution are time-consuming and expensive, limiting their utility in fighting novel pathogens ^19,20^. The currently approved vaccines for SARS-CoV-2 are built upon prior developed SARS-CoV and MERS vaccines. Current vaccines utilize the critical knowledge about the immunopathological effects of targeting the nucleocapsid protein^21,22^, as well as the 2p mutation used to stabilize the spike protein in the most immunogenic form^23^.

Conventional vaccines are composed of whole pathogens or their components. As a result, they can induce unwanted side effects, such as vaccine enhanced infection, interfering neutralization antibodies, Hoskins effect, toxicity, and pathogen de-attenuation^24–28^. These issues put the immunocompromised vaccine recipients at even greater risk and further hinder vaccine development^29^. Additionally, a certain percentage of current vaccine recipients experience cardiovascular complications such as inflammation and myocarditis ^30,31^. With these limitations in mind, we aim to develop a novel vaccinology approach such as a modular epitope vaccine as a proof-of-concept against SARS-CoV-2.

Our strategy is based on short peptide epitopes that generate immune responses precisely aimed at the critical components of the SARS-CoV-2, limiting the adverse effects^32–34^. The immunogen is assembled on a long single-stranded DNA molecule to which short DNA sequences (probes) are annealed in a sequence-specific manner. Each DNA probe carries a specific epitope (peptide) covalently attached to the end of the DNA. We used two peptides corresponding to well-identified linear epitopes of the S-protein on SARS-CoV-2^35^. To endow the vaccine with antigenicity Pan HLA DR-binding Epitope (PADRE), ^36^ was attached to the corresponding DNA probe incorporating it into the immunogen. As a result, the immunogen was obtained by annealing the long DNA template with a mixture of DNA-peptide conjugates. Several formulations of immunogen were made for immunization of mice to evaluate the S-protein and the peptide-specific immune responses. We found that our novel vaccine design generated an immune response in mice without any adverse events, and serum from these mice could neutralize SARS-CoV-2 and its VOCs. This study provides an insight into the applicability of this vaccine platform to SARS-CoV-2 and its VOCs.

## Results

### Validation of neutralizing linear B-cell epitopes from the SARS-CoV-2 S-protein upon conjugation with DNA probes complementary to DNA scaffold.

**Figure 1:**
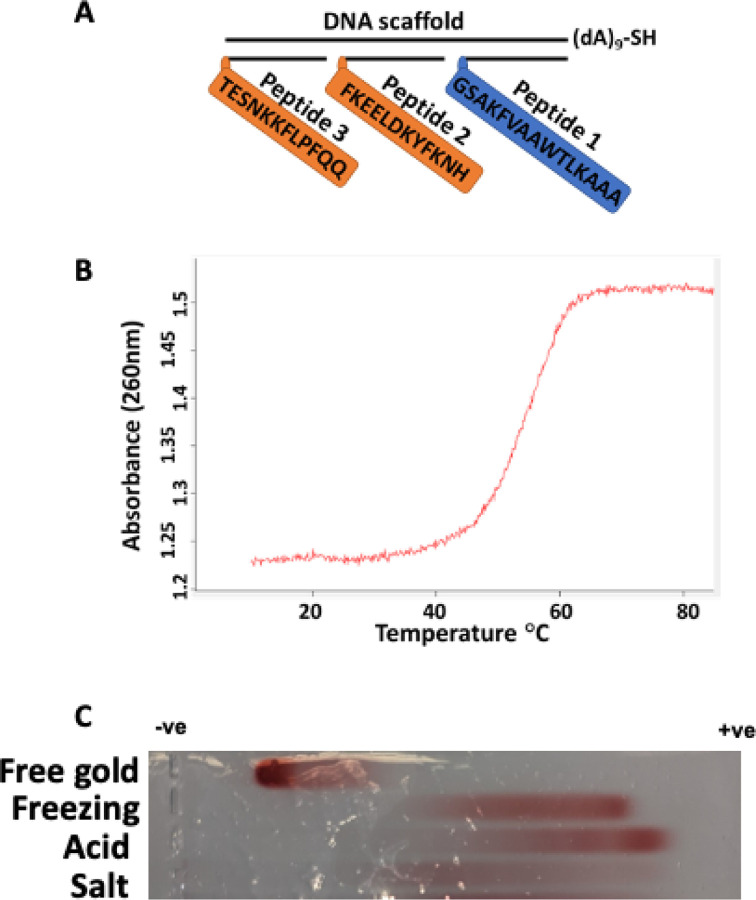
Schematic representation of immunogen and its validation. Three epitopes as peptides (P) 1, 2, and 3 are assembled on a DNA scaffold containing thiol for coupling with gold. According to Fig. S1, P1 is a universal T helper cell epitope PADRE, P2 is he S-protein-derived peptide (residues 1148–1159), important for cell membrane fusion, and P3 is the peptide located after RBD (residues 553–564), important for receptor recognition (A). Immunogen assembly was validated using spectrophotometric absorbance at 260 nm and the melting curve was taken at a temperature range from 10 to 85°C (B). Immunogen-gold formulations generated by different methods characterized by agarose gel electrophoresis. From top to bottom: free gold nanoparticles, freezing-based conjugation of immunogen, acid-based conjugation, salt-based conjugation. (C)

The immunogen is assembled on a long DNA molecule, which acts as a scaffold to which short DNA oligonucleotides (probes) are annealed in a sequence-specific manner (Fig. 1a). Each DNA probe carries a specific epitope (peptide) covalently conjugated to the end. The peptides are selected from reported neutralizing linear B-cell epitopes (peptides 2 and 3) from the SARS-CoV-2 S-protein^35^. These peptides are synthesized chemically, so any needed post-translational modification can be included in the peptide sequence. The DNA-peptide conjugate is annealed to a complementary sequence on the DNA scaffold. Along with these DNA-peptide probes, a DNA conjugate containing the assembly of the T-helper epitope (PADRE, peptide 1) is annealed. The latter plays a critical role in T-helper-mediated immune response initiation and activation. The construct is obtained by annealing the long DNA scaffold with a 1:1 mixture of the DNA-peptide conjugates (peptides 1, 2, and 3) to yield the construct, as shown in Fig. 1a. The validation of the construct assembly as described above was obtained by spectrophotometric melting experiments. The melting profile for this construct is a sharp increase in absorbance around 55°C corresponding to the experimental melting temperature (Fig. 1b). The observed melting temperatures matched the expected melting temperatures predicted theoretically by OligoAnalyzer™ from Integrated DNA Technologies (52–57°C), which confirms the presence of a DNA duplex. The absence of hyperchromic effect at temperatures below 40°C suggests stability at physiological temperature (Fig. 1b).

### Immunogen conjugation to gold nanoparticles (GNP) with FR and PA methods generated particles with a higher density of the immunogen.

Conjugation to gold nanoparticles(GNP) was utilized to improve the uptake and biological stability of the construct^37^. We have used three strategies to conjugate GNP: a) freezing-based conjugation (FR), b) pH-assisted conjugation (PA), and c) salt concentration-based conjugation (SC) as described in the methods section. The fourth formulation is the inulin-based formulation (IN), and it was used as a gold-free alternative since inulin microcrystals are known to improve antigen uptake ^38^. The GNP-immunogen formulations are negatively-charged molecules with the net charge of particles defined by the density of coverage of the nanoparticle by immunogens. Therefore, we used gel electrophoresis to characterize the formulations (Fig. 1c). The immunogen GNP-PA exhibited the highest mobility towards the positive electrode, followed by the GNP-FR formulation, which had slightly lower mobility. The GNP-SC formulation produced a diffuse band with low electrophoretic mobility. Given that the GNP-immunogens mobility is proportional to the coverage of DNA, these results suggested that the immunogen conjugation with FR and PA methods produced particles with a high density of the immunogen compared to the SC method that produced particles with a low density of the immunogen. These validated immunogen formulations were further tested for their ability to induce an antigen-specific immune response in mice.

### GNP-conjugated immunogen by FR and PA methods elicited higher IgG immune response in mice sera against P1 and P2.

**Figure 2:**
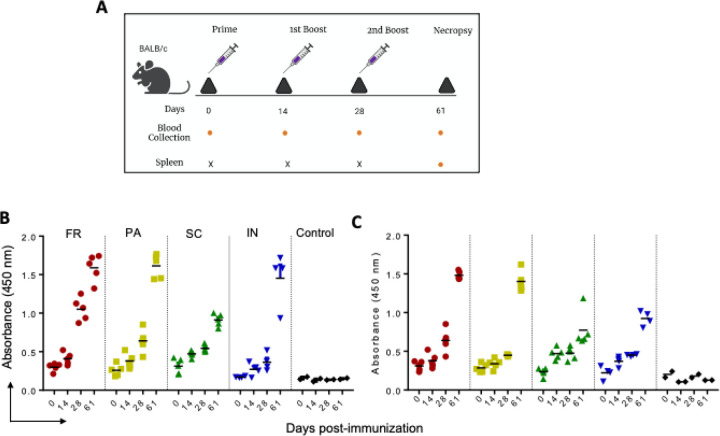
IgG immune response against P1 and P2 in mice immunized with GNP-conjugated vaccine candidates. BALB/c mice were immunized with GNP-conjugated vaccine candidates at different time intervals (Day 0, 14, 28, and 61), blood and spleen were collected as shown in schema (A). High protein binding 96 well plates were separately coated with P1 and P2 (100 ng/well) overnight at 4 °C. Sera prepared from different time points of immunization were diluted and incubated for 1 h at room temperature. IgG immune response against P1 (B) and P2 (C) was determined using HRP-conjugated goat anti-mouse IgG in the presence of TMB as substrate.

Four groups of BALB/C mice were immunized with the validated FR, PA, SC, and IN immunogen formulations (25 mg) with the CpG DNA adjuvant (100ul). Another group of mice received CpG DNA adjuvant alone as a control group. All five groups of mice were immunized with three doses of the respective immunogen as prime dose (0 days), 1st boost (14 days,) and 2nd boost (28 days) as shown in schema (Fig. 2a). Sera were collected at different immunization time points, including necropsy on day 61, and tested for the presence of IgG immune response against peptides 1 and 2 and whole S-protein using ELISA. Plates were coated separately with peptides 1 or 2 (0.1 mg/well) to determine the peptide-specific IgG response. Sera from each time point were tested for IgG immune response starting with the dilution of 1:25 and serially diluted 5 folds up to 1:78125. Interestingly, sera from all the four groups, FR, PA, SC, and IN, indicated the presence of detectable IgG immune response against peptides 1 and 2 at 1:25 on day-61(Fig. 2b & c). However, mice immunized with immunogens formulated by FR and PA methods elicited higher IgG immune response against P1 and P2 with a maximal immune response on day 61. On the other hand, mice immunized with immunogen formulated with IN exhibited a higher IgG immune response against P1 than P2 (Fig. 2b & c). Immunogen prepared with the SC method elicited a lower IgG immune response against both P1 and P2 (Fig. 2b & c). The observed differential IgG immune response by immunogen produced with FR/PA and SC methods correlated with high and low-density immunogen particles (Fig. 1c), respectively. These results indicate that immunogens with high-density particles elicit a higher immune response than immunogens with low-density particles.

### GNP-conjugated immunogen by FR and PA methods elicited higher IgG immune response in mice sera against S-protein.

**Figure 3:**
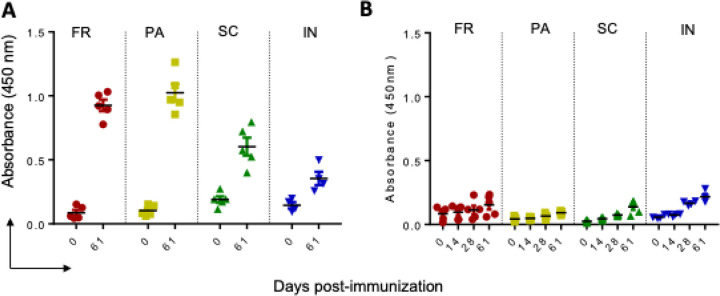
IgG immune response against SARS-CoV-2 whole S-protein and RBD in mice immunized with GNP-conjugated vaccine candidates. High binding 96 well plates were separately coated with whole S-protein and RBD (1 mg/ml) overnight at 4 °C. Sera prepared from different time immunization points were diluted and incubated for 1 h at room temperature. IgG immune response against the S-protein (A) and the RBD (B) was determined, as explained above.

Since the peptides in immunogen formulations are part of the SARS-CoV-2 S-protein, we asked the question of cross-reactivity of IgG immune response against S-protein. The position of the peptides P2 and P3 on SARS-CoV-2 S-protein is depicted in Supplementary Fig. S1. We tested sera from all the groups of mice for IgG immune response against the entire S-protein and, as a control, tested against the Receptor-Binding Domain (RBD) protein. Like IgG immune response against P1 and P2, FR and PA immunogens elicited a higher immune response against the S-protein after the 2nd booster (Fig. 3a). SC and IN immunogens also elicited detectable IgG immune response against the S-protein, although lower than the immunogens formulated by FR/PA methods. Further, to verify antigen-specific IgG immune response, we tested the presence of IgG immune response against RBD of the S-protein. Since peptide epitopes were designed away from the RBD, sera from all the groups of mice failed to react with the RBD (Fig. 3b).

### GNP-conjugated immunogens in mice serum were able to neutralize live SARS-CoV-2 and its VOCs

**Figure 4:**
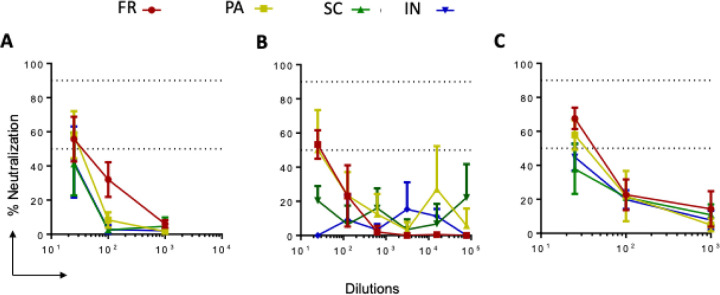
SARS-CoV-2 live virus neutralization ability of sera from mice immunized with GNP-conjugated vaccine candidates. Sera (Day 61) from all groups of immunized mice were evaluated for live virus neutralization ability against SARS-CoV-2 WT, WI, and delta variants. Sera (1:25) were separately incubated with WT, WI, and delta viruses at 10^4^ PFU for 1 h. Following, virus-sera mix was added to the cells and incubated for 24 hrs. Then cells were fixed and permeabilized and stained with the combination of anti-SARS-CoV-2 spike protein antibodies (rabbit mAb) and Alexa Fluor 488 goat anti-rabbit as primary and secondary antibodies, respectively. The nuclei were stained using Hoechst 33342 and plates were read using Operetta Imager. The percent neutralization was calculated based on the differential intensity of the fluorescence.

Since we found a good immune response against peptides and the full-length S protein, we asked whether this was responsible for the neutralization of SARS-CoV-2 and its variants. We pooled sera from all the mice and tested its neutralization capacity against two wild-type SARS-CoV-2 strains. SARS-CoV-2 isolates USA-WA1/2020 (Fig. 4a), SARS-CoV-2 isolate USA-WI1/2020 (Fig. 4b), and one VOC, SARS-CoV-2 isolate hCoV-19/USA/PHC658/2021 (Delta Variant) (Fig. 4c). We found that at 1:25 to 100 dilutions, of sera could neutralize wild type and delta variants more than >50%. (Fig. 4) These data indicate that GNP-conjugated vaccine candidates elicited antigen-specific immune responses with neutralization potential.

### No differential percentage in CD20+ B cells and CD4+/CD8+ T cells memory phenotypes observed across GNP-conjugated vaccine immunized groups.

Using a flow cytometric analysis, we performed a phenotypic assessment of CD4+ and CD8+ T cells and B cells in the spleen, and peripheral blood in control and GNP-conjugated vaccine immunized mice at necropsy. Here, CD4+ and CD8+ T cells phenotypic assessment was performed based on the surface expression of CD44 and CD62L (L-selectin) as memory phenotype and activation markers. The splenocytes and blood cells were stained with fluorochrome-labeled anti-CD4, anti-CD8, anti-CD20, anti-CD44, and anti-CD62L antibodies, and representative gating was used to obtain CD4+ and CD8+ T cells to analyze the expression of CD44 and CD62L (Supplementary Fig. S2). Although gated CD4+ and CD8+ T cells express comparable levels of CD44 and CD62L, no difference was observed in the percentage of naïve (CD44-CD62L+), TCM (CD44+CD62L+), and TE/EM (CD44+CDL-) in splenocytes and blood cells across immunized groups compared to control (Supplementary Fig. S3). Further, splenocytes and blood cells from GNP-conjugated vaccine immunized mice did not exhibit the difference in the percentage of CD20+ B cells compared to control (Supplementary Fig. S4).

## Discussion

This study demonstrated that peptides assembled on the DNA scaffold resulted in an efficient immunogen, producing a strong and specific immune response (Fig. 1A). The formulation of the immunogen is a crucial component for vaccine development. The modular vaccine was assembled by chemical conjugation to gold nanoparticles using three conjugation strategies or co-crystallization with inulin microcrystals. The electrophoresis data (Fig. 1C) suggests that the density of DNA coverage of the gold nanoparticle is the primary factor affecting the immunological efficiency of the GNP-immunogen construct. The thiol-gold coupling method defines the final product’s density, which is consistent with the reported differences^39^. The acidic pH-based conjugation (PA) seemingly produced the highest coverage, closely followed by freezing-based (FR) conjugation. At the same time, the salt concentration-based method (SC) resulted in smearing and low mobility, suggesting low coverage and/or aggregation.

We tested the immunogenicity of the resulting vaccines and found stark differences between formulations. The inulin-based formulation had high anti-Peptide 1 antibody levels and a significantly weaker response to Peptide 2, suggesting dissociation of the immunogen molecule and inefficient immunological synapse formation. Peptide 1 serves as B-cell and T-helper epitope simultaneously and thus can form immunological synapses regardless of formulation and assembly. Salt concentration-based SC formulation had a poor immune response to both peptides, suggesting poor uptake or processing by antigen-presenting cells due to aggregation or loss of antigen during dialysis. Finally, acid-(PA) and freezing-based (FR) formulations had almost identical antibody levels against both peptides P2 and P3, indicating the stability of the formulation.

**Figure 5:**
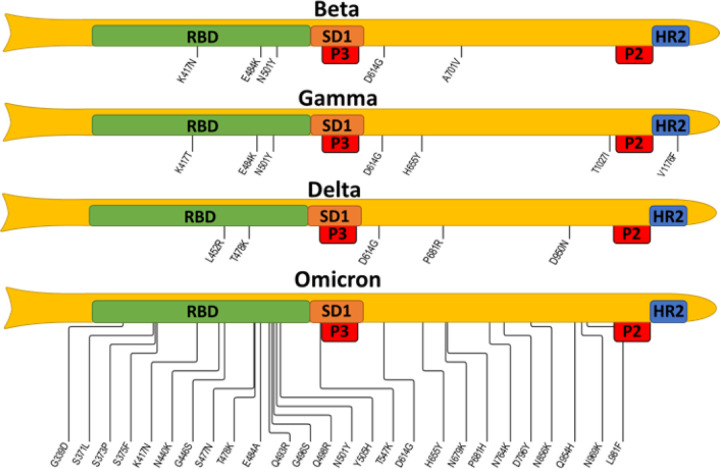
SARS-CoV-2 VOCs map of mutations spanning from RBD to heptad repeat 2 (HR2) The four spike protein variants: beta, gamma, delta, and omicron BA1, are represented schematically; note that the N-terminal domain and mutations localized within are omitted. Selected epitopes are expected to disrupt the activity of the subdomain 1 (SD1) and heptad repeat 2 (HR2), thus blocking viral attachment and cellular entry. The information about mutations is taken from the covdb.stanford.edu database.

Further, the immune response against the full-length S protein or RBD (negative control) confirmed the findings of peptide ELISA, with strong immune responses to S protein in acid- and freezing-based formulations and minimal immune response to RBD. Being only detected in sera generated against PA- and FR-based formulations, which had a high concentration of spike-targeting antibodies and showed neutralization titers against wildtype SARS-CoV-2 and delta variant^40^. Importantly, according to Fig. 5 in which maps of mutation in the currently monitored VOCs are shown, the selected epitopes do not carry any of these mutations This finding suggests that our vaccine should be equally efficient for all current variants including Omicron variant.

The developed peptide-array approach has several important features. The accessibility and affordability of solid-phase peptide synthesis of short peptide epitopes simplify the manufacturing of our vaccine^41^. We use programmed-complementarity DNA-based assembly of separate epitopes into a modular vaccine, which further improves adaptability, simplifies chemical synthesis and formulation. Most epitope vaccines utilize chemical conjugation of the epitopes to a carrier protein, such as ovalbumin, or combine the epitopes into a single fusion protein like beads on a string. Since the epitopes incorporated in such immunogens have no explicit borders, they can go through different patterns of proteolytic processing and produce a variety of new, uncharacterized, and unwanted epitopes, known as neoepitopes ^42,43^. The folding of these immunogens produces additional conformational neoepitopes, complicate storage and result in large batch-to-batch variability, should the artificial protein have several stable structures^44^. The use of rigid DNA duplex for immunogen assembly keeps the epitopes in our vaccine structurally and spatially separated, minimizing the risk of neoepitope formation. Finally, DNA has low antigenicity and does not lead to carrier-induced epitope suppression^45,46^. An epitope vaccine can generate a precise immune response to the neutralizing epitopes with high efficacy, with little or no therapeutically inefficient or harmful antibodies. This is critical when targeting some parts of the pathogen can lead to immune enhancement of the disease or adverse reactions. A well-targeted epitope vaccine allows for protection with minimal stimulation of the immune system^47–50^. before an immunogen to be effective, it must contain the B cell target moieties on the surface and have the T-helper cell epitopes attached to them strongly enough to ensure simultaneous uptake into the immune cells. So far, several studies have reported the critical role of antigen-specific B cells in the development and differentiation of CD4 T cells memory phenotypes^51–54^. T-helper cells in mice can be categorized into memory and naïve phenotypes based on the expression level of CD44 and CD62L (L-selectin). CD44lowCD62L+ population considered as naive, CD44highCD62L+ population considered central memory (TCM), and the CD44highCD62L-population considered effector and/or effector memory (TE/EM)^57,58^. Although there were no differences in the percentage of T-helper memory phenotypes across the immunized group, the functionality of T-helper cells cannot be ruled out.

In conclusion, to our knowledge, this is the first epitope vaccine made through DNA hybridization-based assembly of separate epitopes into a single molecule against SARS-CoV-2. The use of nucleic acids to achieve this is a very convenient strategy since they are non-toxic, easy to synthesize and characterize. Most importantly, they are highly programmable, allowing for reproducible and predictable assembly. Since simple mixing of target epitopes and immunostimulants cannot efficiently deliver them to the antigen-presenting cells and facilitate the formation of an immunological synapse. Our newly developed platform greatly simplifies the fusion of immunomodulatory ligands, such as TLR agonists, allowing for direct delivery of these ligands into the immune cells. The use of fused TLR agonists is known to significantly improve vaccine efficacy^59,60^, allowing for T-cell-independent B-cell maturation^61^, which is important for elderly and immunocompromised patients, all while minimizing side effects effects^62,63^.

## Methods

### Vaccine preparation.

The peptides (N3-Lys)-GSAKFVAAWTLKAAA (peptide 1), corresponding to pan HLA DR-binding epitope PADRE, designed to stimulate T helper cells in genetically diverse populations), (N3-Lys)-FKEELDKYFKNH (peptide 2), corresponding to residues c of the full spike located right before heptad repeat 2 and is essential for the cell membrane fusion, (N3-Lys)-TESNKKFLPFQQ (peptide 3), corresponding to residues 553–564 of the full spike located on the S1, right after the RBD) have been synthesized by Genscript (Piscataway NJ, USA). DNA oligonucleotides (ssDNA) were synthesized using MerMade 12 DNA synthesizer with the help of the standard phosphoramidite chemistry with details provided in the supplement. The following set of oligonucleotides were synthesized: Strand 1 DBCO-TATACAGCCTACTCACTATA; Strand 2 DBCO-TATACTGAGCTAGTCGTATA; Strand 3 DBCO-TATACCTTCATCCTTATATA and a complement strand (SH)-A_9_TATAGTGAGTAGGCTGTATATATACGACTAGCTCAGTATATATATAAGGATGAAGGTATA. Citrate-coated gold nanoparticles 15 nm in diameter and at OD 50 (Luna Nanotech, Markham, ON, Canada) were used for formulation.

### Peptide-DNA coupling.

Each peptide and DNA strand were dissolved in 6 M guanidinium hydrochloride in the presence of sodium phosphate buffer (50 mM, pH 7.0) at the final concentrations of 200 µM ssDNA and 400 µM peptides. The reaction was carried out for 24 h at 4°C, followed by dialysis to remove excess peptides. Reaction completion was verified by the disappearance of 308 nm absorbance shoulder on UV-Vis, corresponding to the unreacted DBCO.

### Assembly of the antigen.

Peptide-coupled DNA strands (1–3) were combined in isotonic 0.5 X PBS pH 7.4 at a concentration of 150 µM and 140 µM of the compliment. The mixture was heated to 95°C followed by gradual cooling to 4°C (2 h). The annealed product was diluted to 1.2 OD at 260 nm in isotonic PBS, and the assembly was verified by the melting experiments using UV-Vis spectrophotometer Varian Cary 50 Bio with a temperature range of 10–85°C, data interval 0.1°C, temperature ramp rate 2°C/minute, signal averaging time 0.1. The melting temperature was compared to the theoretically melting temperature predicted by OligoAnalyzer™ from Integrated DNA Technologies (IDTDNA.com).

### Vaccine formulation.

Vaccine candidates were formulated by freezing-based (FR), pH-assisted (PA), salt concentration-based (SC) conjugations, and inulin co-crystallization (IN). In the FR method, the annealing product was diluted in water 4-fold (<20mM Na^+^) and added to the GNP stock solution (136 nM) at a molar ratio of 150:1. The mixture was placed in a −20°C freezer for 1 h. Then, the product was allowed to thaw, and 10 x PBS was added to adjust the tonicity of the final product to isotonic. The solution was diluted to 50 nM (as GNP) in PBS, aliquoted at 100 µl/dose, and frozen for use as an immunogen.

In the PA method, the annealing product was combined with GNP stock solution at a molar ratio of 50:1 and incubated for 5 min. Then, pH was adjusted to 3.0 with 1M citric acid, incubated for 3 min, and neutralized with NaOH to pH 6.5. Heat annealing was performed as described previously. The conjugate was dialyzed against isotonic PBS, diluted, and aliquoted as in the FR method.

In the SC method, the initial procedure was identical to the FR method. Instead of freezing, saturated NaCl (6.15 M) was gradually added to a 1 M final concentration (48 h). The conjugate was dialyzed against isotonic PBS, diluted, and aliquoted as in the FR method.

For IN co-crystallization conjugation, inulin microcrystals (epsilon form) were prepared as described in ^64^. The microcrystals were washed five times via centrifugation and resuspension in 45°C water. After the final centrifugation, the wet pellet was resuspended in the annealed product at final proportions of 300 mg of wet pellet and 5 moles of annealed product per 1 ml in isotonic PBS. The mixture was then heated up to 55°C and allowed to cool down slowly. The resulting microcrystals were aliquoted at 100 µl/dose and stored at 4°C until further use.

### Agarose gel electrophoresis.

Agarose (2%) (Sigma-Aldrich, Inc. St. Louis, MO) gel was prepared in sodium borate buffer (10 mM, pH 8.0) and used for electrophoresis of unconjugated and conjugated GNP prepared by the FR, PA, and SC methods. The nanoparticles were loaded directly in buffer without loading dye at 10 µl/well, and electrophoresis was carried out at 15 V/cm (300 V).

### Cells and viral titer determination.

Vero E6 (ATCC® CRL-1586™) and Vero-STAT1 knockout cells (ATCC® CCL-81-VHG™) were cultured in DMEM containing 10% fetal bovine serum, 2 mM L-glutamine, penicillin (100 units/ml), streptomycin (100 units/ml), and 10 mM HEPES. Calu-3 cell (ATCC 184HTB-55) were cultured in Eagle’s 188 Minimum Essential Medium (EMEM) (ATCC 30–2003) containing 10% FBS. SARS-CoV-2 isolates USA-WI1/2020 (BEI; cat# NR-52384), USA-WA1/2020 (BEI; cat# NR-NR-52281) were passaged in Vero-STAT1 knockout cells, whereas hCoV-19/USA/PHC658/2021 (Delta Variant) (BEI; cat# NR-55672) was passaged in Calu-3 cells. The viral titer was determined using the plaque assay as described previously ^65^. In brief, Vero E6 cells (2.510 ^5) were seeded in 6-well plates and incubated for 24 h. After 24 h, cells were washed with sterile 1X PBS, and the virus stock was ten-fold serially diluted in serum-free OptiMEM media and then added to the cells in duplicate. The plates were incubated at 37°C for 1 h with slight shaking every 15 minutes. Then, 2 ml of 0.5% agarose in minimal essential media (MEM) containing 5% FBS and antibiotics were added to each well and incubated at 37°C for 72 h. The cells were fixed with 4% paraformaldehyde overnight, followed by removing the overlay and staining with 0.2% crystal violet to visualize plaque-forming units (PFU). All assays were performed in a BSL-3 laboratory setting.

### Animal Experiments

BALB/c mice (males, 6 to 8 weeks old) were purchased from Jackson Laboratories and housed in micro isolator cages and maintained at 12-h light-dark cycle at 22.2°C, and 30–40% humidity. Mice were given feed and sterile water daily, and they were acclimated to the environment for approximately 1–2 weeks to determine that they were healthy and suitable for experiments. Four groups of mice (n=5 each group) were immunized subcutaneously with GNP-conjugated vaccines (100 µl) by different methods, FR, PA, SC, and IN. The immunogens were diluted in LPS free water and mixed with 25 µg ODN 1826 Class B CpG oligonucleotide (a murine TLR9 ligand) as an adjuvant. Another group of mice immunized with only 25 µg ODN 1826 Class B CpG oligonucleotide serves as the control. Baseline bloods were collected from the submaxillary vein on day 0 (prior to immunization). Blood samples were collected every 14 days with boosts of the same doses were given. All mice were immunized with 2 boosts and finally necropsied at day 61. Blood samples and spleens were collected at necropsy for flow cytometry. All the animal procedures, including housing, are approved by the University of Nebraska Medical Center (UNMC) Institutional Animal Care and Use Committee (IACUC) protocol # 21–020-04-EP. and are conducted according to the National Institutes of Health guidelines. Furthermore, this study is reported in accordance to ARRIVE guidelines (https://arriveguidelines.org/arrive-guidelines).

### Enzyme-Linked Immunosorbent Assay (ELISA).

Ninety-six-well high binding plates were separately coated with peptide P1, P2 (100 ng/well), whole S-protein, and spike-RBD protein (0.1mg/well) 1X PBS and incubated 4°C overnight. The next day, the plates were washed three times and blocked with 1% BSA in 1X PBS containing 0.1% Tween 20 (PBST) for 1 h at 37°C. Sera samples were diluted starting from 1:40 (please check this) and five-fold serially in 1% BSA containing PBST were added to the plates and incubated at 37°C for 1 h. The plates were washed three times with PBST and HRP-conjugated goat anti-mice IgG Human ads-HRP (SouthernBiotech1:4,000 dilution) (cat#103005) was added and incubated for 1 h at room temperature. Plates were washed five times with PBST before the addition of 3,3′,5,5′-tetramethylbenzidine (TMB) substrate solution. The reaction was stopped after 5 min by the addition of 0.16 M sulfuric acid to stop the reaction. The OD at 450 nm was measured with a Bio-Rad microplate reader. A graph was plotted as Absorbance at 450 nm vs dilutions.

### Immunofluorescence based Neutralization assay.

Neutralization assays were performed against USA_WI/2020, USA-WA1/2020 and hCoV-19/USA/PHC658/2021 strains. Sera samples were serially diluted (5-fold) in serum-free Dulbecco’s modified Eagle’s medium (DMEM) in triplicate wells and incubated with 20000 focus-forming units of SARS-CoV-2 virus at 37°C for 1 h. The serum-virus mixture was added to Vero E6 cell (C1008, ATCC, no. CRL-1586) monolayers seeded in 96 healthy blackout plates and incubated at 37°C for 1 h. The inoculum was removed and replaced with complete DMEM and incubated at 37°C for 24 h. After 24 h, DMEM was removed, cells were washed twice with PBS and fixed with 4% paraformaldehyde in PBS for 30 min at room temperature. Following fixation, plates were washed thrice with 1X PBS and permeabilized with 50 µl/well of 0.1% Triton X-100 (Fisher BP151–100) in PBS for 10 min. After permeabilization, cells were washed thrice with 1XPBS and blocked with 3% BSA for 30 mins. Then, cells were stained with 50 µl/well of primary antibody, anti-SARS-CoV-2 spike (rabbit mAb, Sino Biologicals MA14AP0204) at 1:1000 diluted in 3% BSA-PBS and incubated overnight at 4°C on a shaker. The next day, cells were washed three times with 1X PBS and stained with a secondary antibody (Alexa Fluor 488 Goat anti-rabbit) at 1:2000 dilution in 3% BSA-PBS. Finally, cells were incubated at room temperature in a shaker for 1 h, washed three times with 1X PBS, and the nuclei were stained using Hoechst 33342 (Invitrogen H3570) and Cell Mask (Invitrogen C10046) at 1:20000 diluted in 1X PBS. The plate was shaken for 15 mins after proper sealing with aluminum foil/sealer and taken for reading using Operetta Imager. Percentage neutralization was calculated based on the difference in fluorescent intensity.

### Analysis of B cells and CD4+/CD8+ memory phenotypes by flow cytometry.

Blood samples and spleen tissues were collected from immunized mice at necropsy. Single-cell suspension from spleen tissues was prepared, and RBCs were lysed using RBC lysis buffer. Cells (5x10^6^) were washed and suspended in staining buffer, and Fc block was performed with purified anti-mouse CD16/32. Dead cells were discriminated by adding Zombie Aqua fixable viability dye. Fluorochrome-labeled mouse-specific antibodies were diluted with staining buffer into cocktails and added to cells at 100 µl per sample. Cells were incubated for 30 min at room temperature and washed twice with staining buffer. Then, cells were fixed using 2% PFA and acquired using the Becton Dickson Fortessa X450 flow cytometer. Fluorescence minus one (FMO) control was performed in parallel, and subsequent gating was determined by FMO. All data were analyzed using FlowJo software

## Statistical analysis

Bars and error bars represent mean G standard error. Data were analyzed using GraphPad Prism (v6.07).

## Supplementary Material

Supplement 1

Supplement 2

Supplement 3

Supplement 4

## Data Availability

All the data supporting the findings of this study can be found within the paper in Figs. 1–5 and in Supplementary data Figs. 1 to 4.
