# Supplementary figures and images for: Peptide Nanoarray Scaffold Vaccine for SARS-COV-2 and Its Variants of Concerns
Source: Res Sq. 2022 Jan 24:rs.3.rs-1206402. Preprint. [Version 1] doi: 10.21203/rs.3.rs-1206402/v1 (PMC8811944; doi:10.21203/rs.3.rs-1206402/v1)

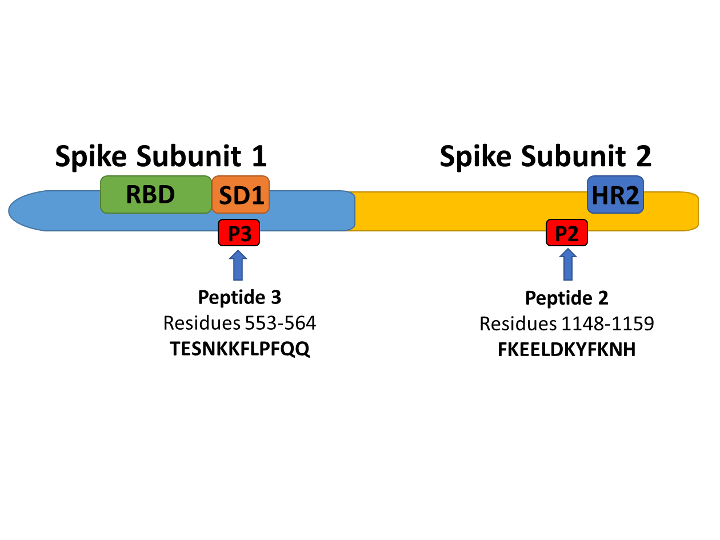

Supplement: Supplement 1 [file 6aea50bdfc7a397e304b8cc4.tiff]

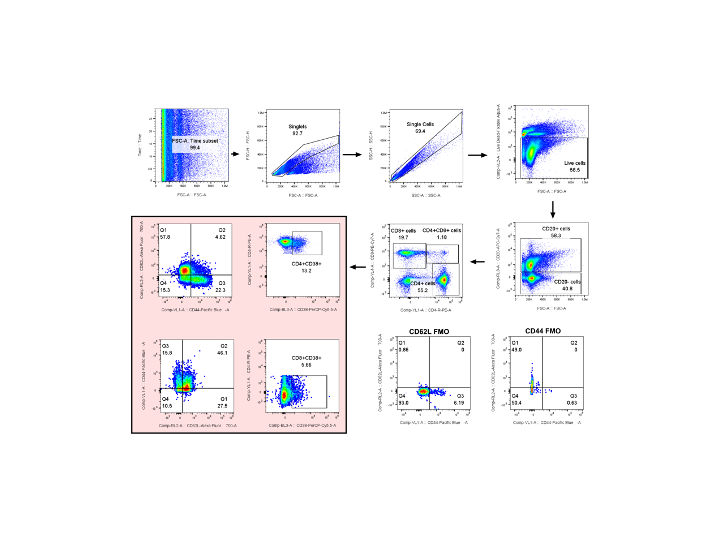

Supplement: Supplement 2 [file c4bfcd6f77d220105411e52f.tiff]

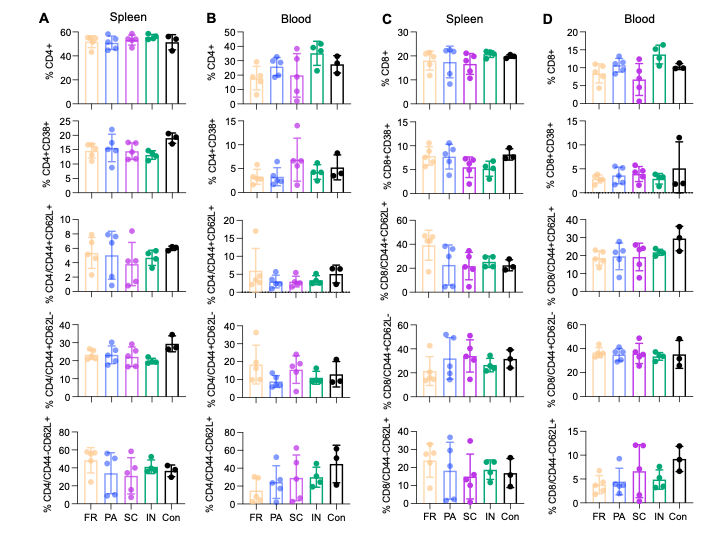

Supplement: Supplement 3 [file ad5069766865a051ea6f5761.tiff]

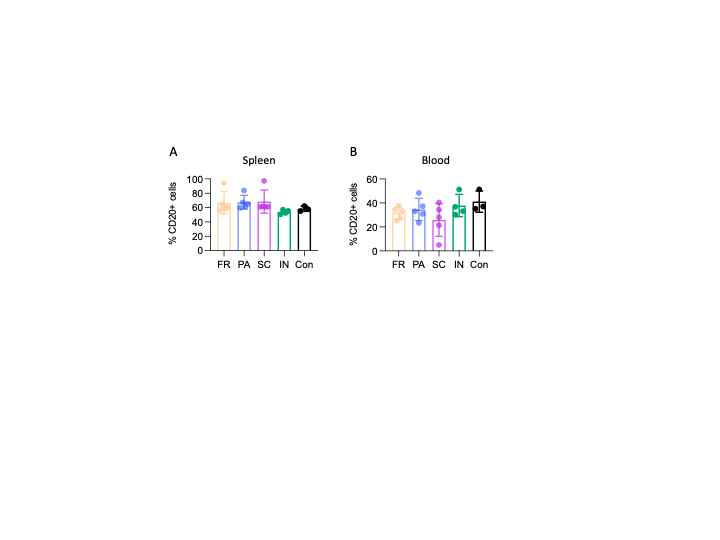

Supplement: Supplement 4 [file dce9a0cef591e292a10cbe06.tiff]
